# Real-Time Peer-to-Peer Observation and Feedback Lead to Improvement in Oral Presentation Skills

**DOI:** 10.7759/cureus.21992

**Published:** 2022-02-07

**Authors:** Rabia Qaiser, Patrick Fadden, Salem Rustom, Jawaid Shaw

**Affiliations:** 1 Internal Medicine, Virginia Commonwealth University School of Medicine, Richmond, USA; 2 Internal Medicine, Hospital Medicine, Hunter Holmes McGuire Veterans Administration Medical Center, Richmond, USA; 3 Biostatistics, Virginia Commonwealth University, Richmond, USA; 4 Internal Medicine, Hospital Medicine, Virginia Commonwealth University School of Medicine, Richmond, USA

**Keywords:** skills, oral presentations, peer-to-peer, curriculum, medical students

## Abstract

Background

Oral case presentation is a vital skill in many fields, particularly in medicine, and is taught early on in medical schools. However, there is a diminished focus on the development of this skill during the clinical years. In this study, we investigated whether the implementation of a formal teaching strategy during the internal medicine clerkship rotation can lead to an improvement in oral presentation skills.

Methodology

Students received an introductory PowerPoint lecture and saw brief video presentations summarizing the key components of a successful oral presentation. Subsequently, students were asked to evaluate their peers while they presented during morning rounds using a standardized feedback form in the first and the second half of their rotation. Using the information gained from the feedback form, students provided verbal feedback on the quality of oral presentations to their peers.

Results

A total of 64 students participated in this curriculum at a university-affiliated teaching hospital, and a total of 409 evaluations were completed. The average total score during the first and the second rotation period was 93.0% (standard deviation, SD = 9.8) and 96.9% (SD = 7.1), respectively. Improvement in the total score of 3.7% points was seen in the entire cohort, with an average improvement of 64% (or 1.64 times) in the probability of obtaining a full score during the second rotation.

Conclusions

Our data show improvement in scores between collection blocks using an educational strategy. This study emphasizes the fact that peer-to-peer evaluations helped in the refinement of oral presentation skills.

## Introduction

Oral presentation is a skill that requires concerted effort and practice to serve its desired goal. However, it remains an understudied area in the teaching curricula [1]. Although there is no universal definition of oral case presentation, some key characteristics of high-quality presentations in internal medicine have been described [2,3]. While most students are aware of the standards for oral presentations at times they struggle with execution. Students can also have a different perception compared to their attendings regarding the goal of oral presentation. While the former view the activity as a data collection task allowing information to be delivered for interpretation, the latter view it as an opportunity for constructing an argument for or against specific diagnoses and management [4]. However, the potential advantage of peer-to-peer feedback is that it is informal and occurs in a nonjudgemental manner compared to the attending feedback where such pressures may be perceived by students.

Furthermore, feedback and expectation regarding oral presentations are nonspecific and variable at times. This lack of implicit expectations and varying attending preferences confuse medical students and delay the acquisition of skills [4]. The available literature supports the notion that most students learn by trial and error, suggesting that transparent expectations should be emphasized [4]. Peer-to-peer feedback for improvement of oral presentation competencies has been suggested with potential gains in providing and receiving feedback among students and alleviating some feedback duties for the attending [1]. In the case of teaching attendings, peer observation has recently been shown to lead to improvement in teaching behaviors [5]. However, there is sparse data assessing the use of student peer-to-peer evaluation of the oral presentations carried out during morning rounds. We developed a curriculum for internal medicine clerkship students that emphasizes this skill development as an active learning process along with its improvement through peer-to-peer feedback in real-time.

In this study, our primary objective was to examine whether student-to-student observation and feedback can lead to improvement in oral presentation skills in the inpatient setting.

## Materials and methods

**Figure 1:**
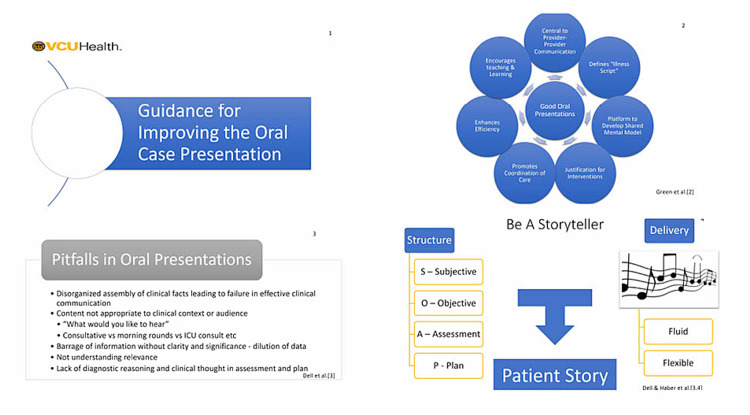
PowerPoint slides (1-4) used in the initial orientation.

**Figure 2:**
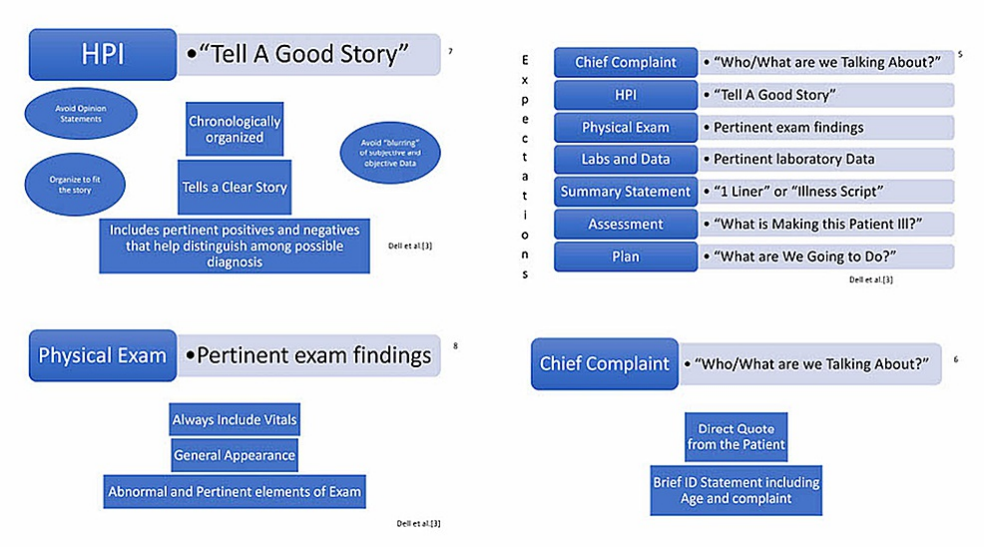
PowerPoint slides (5-8) used in the initial orientation.

**Figure 3:**
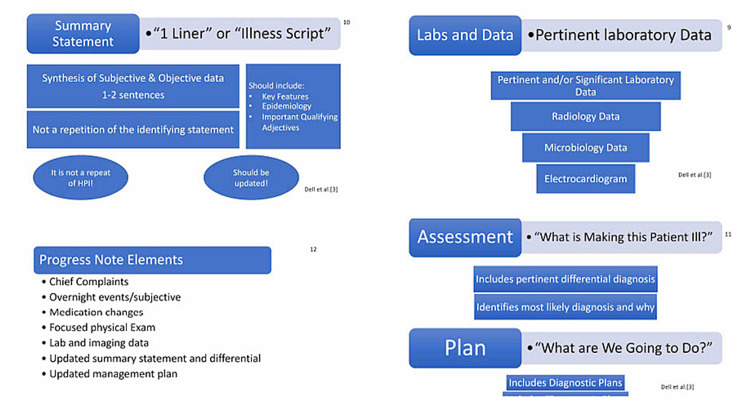
PowerPoint slides (9-12) used in the initial orientation.

This study was conducted at a university-affiliated tertiary care teaching hospital. Our local Institutional Review Board determined that our study was a quality improvement (QI) educational research and was exempt from full approval. The internal medicine clerkship is an eight-week rotation, with four continuous weeks completed at the study hospital. The curriculum was implemented from April 2018 through December 2018. At the beginning of the rotation during the orientation session, the students were given a talk titled “Guidance for Improving Oral Case Presentation.” This talk was delivered using a PowerPoint presentation prepared by the investigators along with a showing of the two selected educational resource videos on the topic (poor presentation and good plan presentation) to highlight the contrast between a well-narrated and a disorganized presentation from the reference material [6]. The presentation aimed to explain and emphasize the elements of a good and successful oral presentation and the common pitfalls thereof. This talk helped introduce the curriculum to the students and set their expectations. Overall, an average of 40-50 minutes were spent on this effort, followed by answering any questions students had. Figures 1-3 show the PowerPoint slides used in the initial orientation [6].

Peer-to-peer evaluation tool and process of peer observation

**Figure 4:**
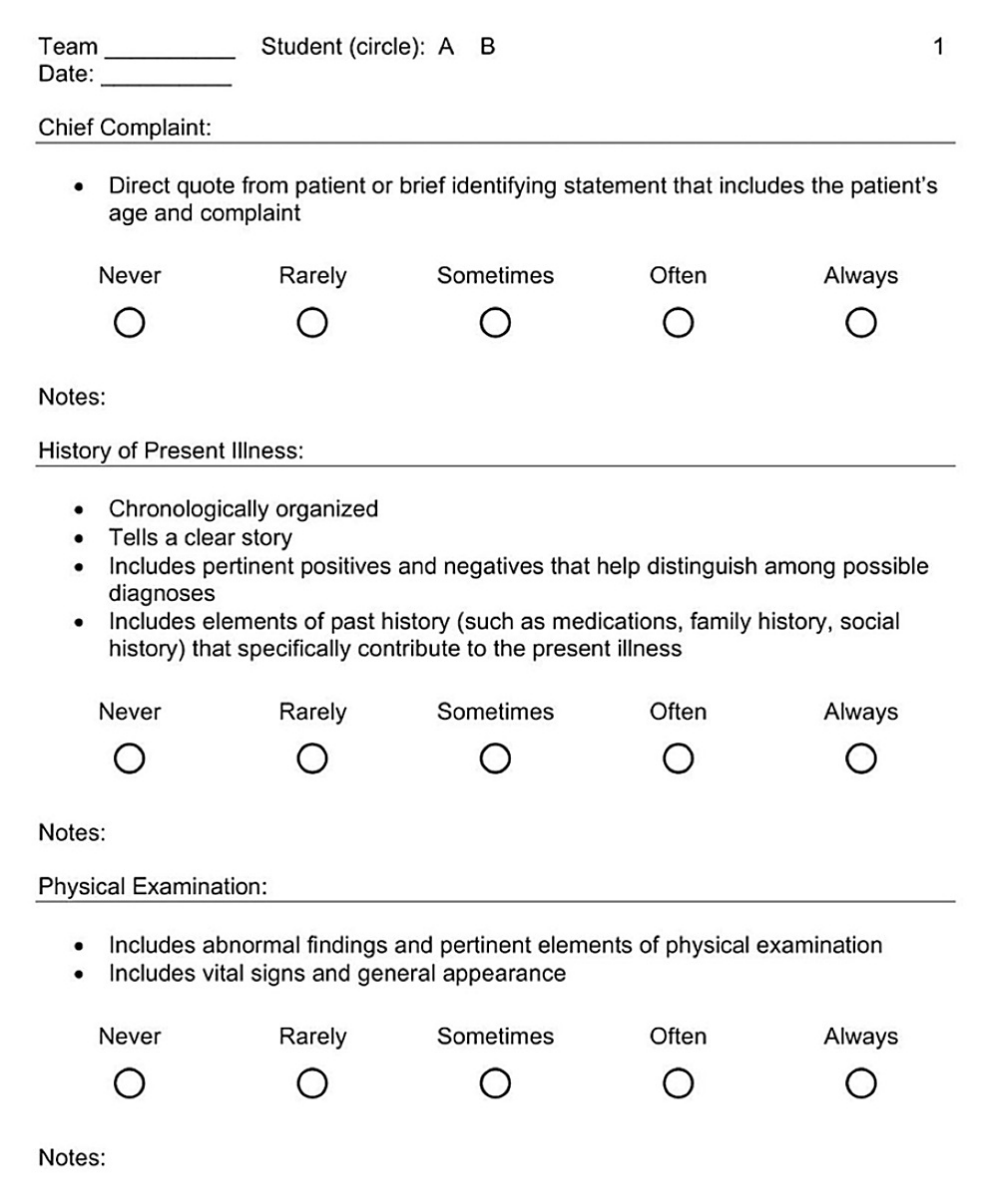
Student peer-to-peer evaluation tool (page 1) used in our study.

**Figure 5:**
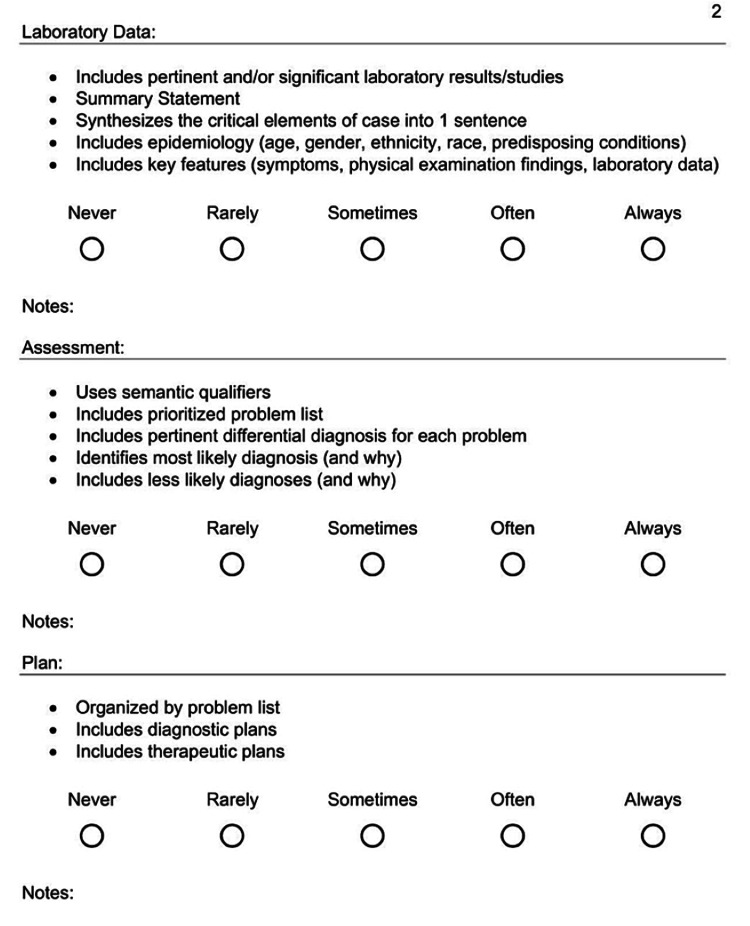
Student peer-to-peer evaluation tool (page 2) used in our study.

The students were divided into four groups which differed by their rotation schedule. Group 1 involved students who were performing their clinical rotation for the first time (least experienced), while Group 4 had experience with oral presentations from their previous clinical rotations (most experienced). We used the domains for oral presentation as laid down and validated in earlier studies for our student peer-to-peer evaluation tool [3,7]. However, we added a Likert scale to score the tool, ranging from 1 (never) to 5 (always), where 5 was considered the best possible desired outcome for that particular metric. The following six different metrics were evaluated: chief complaint, patient illness history, physical examination, laboratory data, assessment, and plan. In addition, space for free comments was left for each of the metrics, and the total number of comments for each presentation was counted at the end. Figures 4, 5 show the student peer-to-peer evaluation tool used in this study.

During the first two-week rotation, students were given six evaluation forms to assess their peers’ presentation performance. Another set of identical evaluations was provided for their second two-week rotation. They were encouraged but not mandated to fill out all the forms. Evaluation sets were marked by team number, and then within each team, evaluations were labeled as “Student A” or “Student B.” Student pairs self-assigned one another as “Student A or B” and kept this designation consistent throughout four weeks. This was done to maintain the anonymity of the students for the investigators.

Each student would listen to their peer’s oral presentation in the morning and fill out the evaluation form at the same time. They were later expected to provide oral feedback to each other once the round was finished based on their evaluation of the forms filled out earlier. This feedback activity was neither coached nor witnessed by the teaching attending on the teams. Each week students were anticipated to complete between one to three sessions of giving and receiving feedback per student. Because the student participation in these evaluations was voluntary, the number of returned evaluations per student was not the same, even within the same presenter during different rotation periods. Thus, proportions (the obtained score divided by the total possible score by period) rather than just the obtained score were used for the outcomes.

Statistical analysis

The abovementioned six metrics and the differences between the latter and initial rotation were summarized by the mean and standard deviation (SD). The outcomes were summarized all together and by group and criterion. The number of comments was summarized by the median and interquartile range due to the extreme skewness in the distribution. Due to the small sample size, preventing model convergence of a (frequentist) beta mixed model, Bayesian beta mixed models with random intercepts were performed for each of the six outcomes independently, as well as to assess the differences between rotations after adjusting for group membership. Because some observations had full scores, the Lemon-Squeezer transformation was used to slightly shift the outcome so that full score observations could be handled by the beta mixed model. This transformation y' is defined as y' = [y(N - 1) + ½]/N, where y is the outcome of interest and N is the sample size [8]. Using Markov Chain Monte Carlo (MCMC) sampling, 10,000 observations were used for the burn-in period, followed by sampling 20,000 observations. Model convergence was assessed via trace plots and the multivariate potential scale reduction factor (PSRF). Based on the trace plots and multivariate PSRF, all the Bayesian beta mixed models converged. The mean of the conditional posterior distribution of odds ratios (multiplicative increase in the probability of obtaining a perfect score) along with 95% credible intervals was provided for each of the outcomes.

Posterior distributions summarize what we know about a parameter (i.e., odds ratios) by combining prior knowledge with information obtained from the data in the current study. Sampling from the posterior distribution (in this case, via MCMC sampling) forms the basis of most kinds of practical Bayesian Inference today. Thus, the posterior mean is the mean of the (20,000) samples after burn-in. Credible intervals (Bayesian) are analogous to confidence intervals used in frequentist statistics but have the advantage of having a more intuitive interpretation than confidence intervals. For credible intervals, it can be said that: “The probability the parameter of interest (i.e., odds ratios) falls within the credible interval is 95%” rather than “If we were to repeat the study over many times we would expect on average the parameter to fall within the confidence interval 95% of the time.” Observations with missing data were omitted from the analysis. The statistical analysis was performed using R version 3.6.1.

## Results

A total of 64 students were evaluated on their presentations, with each having anywhere between one to six peer evaluation forms filled out per presentation. Table 1 summarizes the six outcomes in each group and the differences between the first and second weeks of rotations.

**Table 1:** Summary of evaluation scores by criteria and group. ^a^: Missing; n = 1; ^b^: Missing; n = 2; ^c^: Missing; n = 6; ^d^: Missing; n = 8. SD: standard deviation; IQR: interquartile range

	All criteria, Mean % (SD)	Chief complaint, Mean % (SD)	History, Mean % (SD)	Physical examination, Mean % (SD)	Laboratory data, Mean % (SD)	Assessment, Mean % (SD)	Plan, Mean % (SD)	Comments, Median (IQR)
All groups (n = 64)								
First time	93.0 (9.8)	94.1 (10.3)	92.6 (9.2)	96.3 (7.7)	93.1 (9.4)	90.0 (10.8)	92.0 (10.5)	2.0 [0.0, 7.0]
Second time	96.9 (7.1)	95.4 (10.2)	97.5 (6.6)	97.5 (6.3)	96.6 (6.0)	96.1 (7.8)	98.2 (4.5)	1.0 [0.0, 6.0]
Difference	3.7 (11.3)	1.7 (13.5)	4.8 (11.9)	0.4 (9.3)	3.5 (9.4)	5.8 (12.0)	5.9 (10.6)	0.0 [-1.0, 1.0]
Group 1 (n = 11)								
First time^a^	93.9 (8.0)	95.4 (5.55)	93.6 (8.48)	95.0 (10.01)	95.4 (5.97)	92.2 (8.70)	91.6 (8.97)	2.0 [0.0, 4.0]
Second time^c ^	93.5 (11.0)	91.0 (15.24)	93.0 (13.37)	93.0 (10.59)	94.0 (6.99)	92.0 (13.17)	98.0 (4.22)	0.5 [0.0, 1.0]
Difference	0.4 (14.3)	-5.6 (16.43)	0.56 (16.86)	-1.5 (17.33)	-0.9 (7.37)	2.1 (14.78)	7.9 (10.07)	-1.0 [-1.0, 0.0]
Group 2 (n = 14)								
First time^b^	92.3 (11.0)	90.4 (14.98)	92.0 (9.61)	99.0 (3.58)	90.7 (11.32)	89.5 (10.52)	92.0 (11.62)	4.0 [0.0, 11.5]
Second time	98.5 (4.7)	98.3 (5.17)	98.5 (3.22)	98.8 (5.00)	96.5 (6.60)	98.8 (5.00)	100 (0.00)	2.5 [0.0, 6.3]
Difference	6.1 (9.7)	7.7 (11.41)	6.8 (9.21)	-0.5 (1.76)	5.2 (10.58)	9.0 (8.31)	8.0 (11.62)	0.0 [-0.8, 1.0]
Group 3 (n = 6)								
First time^b^	93.8 (9.3)	98.2 (4.60)	92.7 (8.42)	94.8 (8.34)	94.1 (8.82)	89.9 (11.40)	93.0 (11.96)	3.5 [1.3, 16.0]
Second time^d^	97.0 (5.9)	93.1 (10.02)	97.3 (3.56)	99.6 (1.17)	99.6 (1.17)	95.0 (7.56)	97.5 (4.63)	7.0 [5.0, 12.8]
Difference	0.5 (9.6)	-5.0 (13.15)	1.2 (9.76)	2.9 (7.56)	3.8 (7.56)	-0.5 (12.54)	0.5 (5.60)	2.0 [-3.0, 4.5]
Group 4 (n = 16)								
First time	92.2 (10.7)	92.5 (11.60)	92.1 (10.74)	96.6 (7.34)	92.1 (10.92)	88.6 (12.73)	91.5 (10.45)	1.0 [0.0, 4.8]
Second time	97.4 (6.0)	96.5 (10.15)	99.5 (1.37)	98.0 (4.32)	96.8 (6.03)	96.7 (4.71)	96.7 (6.20)	0.0 [0.0, 6.3]
Difference	5.1 (10.6)	3.9 (11.10)	7.4 (11.21)	1.4 (7.24)	4.7 (10.09)	8.1 (11.97)	5.2 (11.49)	0.0 [-0.3, 0.3]

The average total score during the first rotation period was 93.0% (SD = 9.8), and it was 96.9% (SD = 7.1) during the second period. There was an improvement in the total cohort score of 3.7 percentage points (SD = 11.3). During the initial presentation, students on average performed best in “Physical Examination” with a mean score of 96.3% (SD = 7.7). In the latter presentation, students overall performed the best on “Plan” with a mean score of 98.2% (SD = 4.5). Therefore, “Plan” had the largest improvement overall with 5.9 percentage points (SD = 10.6), while “Physical Examination” had the smallest improvement overall with 0.4 percentage points (SD = 9.3). Table 2 provides the mean odds ratios from the posterior distribution and their 95% credible intervals in the total score obtained between the two periods overall and for each of the six criteria.

**Table 2:** Posterior mean odds ratios between presentation periods with 95% credible intervals. Bold indicates that the posterior mean odds ratio does not contain 1.

Outcome	Posterior mean	95% credible interval
All criteria	1.64	(1.17, 2.35)
Chief complaint	1.16	(0.79, 1.70)
History	1.49	(1.05, 2.11)
Physical examination	0.99	(0.70, 1.47)
Laboratory data	1.37	(0.96, 2.03)
Assessment	1.79	(1.24, 2.66)
Plan	1.63	(1.15, 2.33)

The odds ratio in a beta regression is interpreted as the multiplicative factor of change in the probability of obtaining a full score in the latter presentation period compared to the first presentation period. Hence, there was a noticeable average improvement of 64% (or 1.64 times) overall in the probability of obtaining a full score in the latter presentation period compared to the first presentation period. “Patient Illness History,” “Assessment,” and “Plan” also showed noticeable improvement with a 49%, 79%, and 63% increase, respectively.

There were different patterns of change in the score when analyzed by the group. While “Plan” and “Patient Illness History” consistently improved between the four groups, others like “Chief Complaints,” “Physical Examination,” and “Laboratory Data” do not as some groups do worse in the latter presentation period. Table 1 shows that the patterns of improvement or worsening are not consistent across groups. Groups 1 and 3 have a very high level of missingness (43.8% and 62.5% missing, respectively), while Group 2 has some missingness (12.5%), and Group 4 has no missing observations. Groups 1 and 3 show little improvement overall (0.4 and 0.5 percentage points, respectively), while Groups 2 and 4 show some improvement (6.1 and 5.1 percentage points, respectively). As shown in Table 1, there is little overall difference in the number of comments between the two instances of presentation for all groups. The largest increase in comments between groups was in Group 3 with a median difference of 2.0 comments, while Group 1 had fewer comments in the latter presentation (median difference of 1). While there were some presentations with over 20 comments, the majority had little to none. Overall, there were a total of 524 comments recorded by students.

## Discussion

Our study shows performance improvement in the oral presentation when comparing peer-to-peer evaluations in the last two weeks to the first two weeks of the rotation. This lends credence to the suggestion that a formal peer-to-peer evaluation curriculum during internal medicine clerkship may facilitate improvement in oral presentation skills. We designed our curriculum to empower students to understand that the clinical oral presentation is a true skill that required continued practice and edition. Hence, this should not be left to the traditional “trial and error” process. This was done by promoting peer-to-peer feedback which remains an unexploited source of feedback in medical school education. However, this process is successfully being utilized in nursing education [9].

The improved performance seen could be secondary to active participation by students as they were becoming more mindful about the evaluation and feedback. In the process of peers observing each other, they become more active listeners rather than “zoning out” when not presenting themselves on the rounds. Hence, active listening results in improved performance. Again, this awareness of being observed by their peers led to enhancement in “Patient Illness History,” “Assessment,” and “Plan.” As a learning tool, reflection has been shown to result in the acquisition of new and lasting skills, and we think that by implementing our curriculum, we are using the same concept [10].

Currently, there is a lack of a good model that projects the rhetorical and linguistic skills to produce and deliver a good presentation [11]. Investigators have attempted a nonconventional curriculum (video recordings) for the acquisition of oral presentation skills by students [1]. When medical students were given videography-assisted feedback on oral case presentations, it led to significantly reduced anxiety compared to those not getting filmed or receiving any feedback [12]. Our study is different in that we tasked students to evaluate their peers while they were presenting during morning rounds. Our intention for the implementation of the curriculum is not to absolve the faculty of the feedback duties. Instead, we aim to augment the feedback experience for students, leading to their accelerated growth. However, for student peer-to-peer evaluation to reach the level of independence and comfort, it will need continued practice. For persuasive presentations, students need to get a grasp of the medical rhetoric and realize that the use of relevant language is an art that needs to be developed over time. We do not agree any less with the statement, “a great presentation requires style as much as substance; your delivery must be succinct and smooth” [13].

Some of the representative comments were as follows: “good job getting a direct quote from the patient,” “great flow of the history of present illness,” “includes pertinent elements of the history,” “great job summarizing all the lab data,” and “definitely was able to propose differentials pertinent to exam findings,” among others. Most comments were taken from the general guidelines on the feedback form and rewritten. The use of words such as “great” or “good” job is very common, which leads us to believe that providing specific, constructive feedback is a difficult task. One measure could be to formalize this expectation, which may improve compliance and increase the impact of the feedback. Students may benefit from a “de-briefing” at the end of their rotations to share any gained insights and experiences. We firmly believe that learning in a real-time setting led to enhanced outcomes compared to a simulated situation. It appears that one of the barriers to obtaining more accurate results could be secondary to inflated feedback students gave to each other. This may be secondary to students not wanting to face retaliation or ruin a working relationship with their peers. The physician feedback process is a lifelong endeavor learned by trial and error. Hence, early awareness of the importance of this subject through small group discussions or the “two stars” and “one wish” feedback method may be one way to implement this type of learning [14].

Due to the small sample size, it was not possible to assess (via statistical inference) the differences in improvement over time between groups. The excessive missingness (>50% missing) in Group 3 was also concerning and can lead to biased results due to deriving inferences from a smaller sample size. A strong assumption of missing completely at random (MCAR) was made as opposed to missing not at random (MNAR) because the missing observations were ignored. If the mechanism of missing data was MNAR (e.g., missing evaluations due to performing poorly), then there will be a positive bias in results that would inflate the effect of the “educational talk.” Because the peer evaluation sessions were not witnessed, some evaluation sets only had perfect scores, calling into question the credibility of these scores.

Some of the potential limitations of our study may be that it is difficult for peers to provide more constructive or “negative” feedback at times, which can easily bias the results. However, with time and practice, this can get better. We cannot show causation of oral communication improvement from delivering the educational talk during orientation because we did not include a control group. Finally, a small sample size from one center only cannot allow the generalizability of our results.

## Conclusions

Oral presentation performance improved during the last two weeks compared to the first two weeks of clinical rotations. Our findings set the stage for the consideration of implementing a formal curriculum during internal medicine clerkship, which will lead students to be more mindful about their oral presentation skills. The underexploited peer-to-peer evaluation tool can emerge as the main component of such a curriculum. A further area of exploration would be to assess if the improvement seen in peer-to-peer observation correlates with a discernible improvement seen by the teaching attendings.
